# Understanding stress-induced disorder and breakage in organic crystals: beyond crystal structure anisotropy†

**DOI:** 10.1039/d1sc03095g

**Published:** 2021-10-20

**Authors:** Gabriela Schneider-Rauber, Mihails Arhangelskis, Wei-Pin Goh, James Cattle, Nicole Hondow, Rik Drummond-Brydson, Mojtaba Ghadiri, Kushal Sinha, Raimundo Ho, Nandkishor K. Nere, Shailendra Bordawekar, Ahmad Y. Sheikh, William Jones

**Affiliations:** Department of Chemistry, University of Cambridge Cambridge CB2 1EW UK wj10@cam.ac.uk; Faculty of Chemistry, University of Warsaw 1 Pasteura Street Warsaw 02-093 Poland; School of Chemical and Process Engineering, University of Leeds Leeds LS2 9JT UK; Process Research and Development, AbbVie, Inc. North Chicago IL USA

## Abstract

Crystal engineering has advanced the strategies for design and synthesis of organic solids with the main focus being on customising the properties of the materials. Research in this area has a significant impact on large-scale manufacturing, as industrial processes may lead to the deterioration of such properties due to stress-induced transformations and breakage. In this work, we investigate the mechanical properties of structurally related labile multicomponent solids of carbamazepine (CBZ), namely the dihydrate (CBZ·2H_2_O), a cocrystal of CBZ with 1,4-benzoquinone (2CBZ·BZQ) and the solvates with formamide and 1,4-dioxane (CBZ·FORM and 2CBZ·DIOX, respectively). The effect of factors that are external (*e.g.* impact stressing) and/or internal (*e.g.* phase transformations and thermal motion) to the crystals are evaluated. In comparison to the other CBZ multicomponent crystal forms, CBZ·2H_2_O crystals tolerate less stress and are more susceptible to breakage. It is shown that this poor resistance to fracture may be a consequence of the packing of CBZ molecules and the orientation of the principal molecular axes in the structure relative to the cleavage plane. It is concluded, however, that the CBZ lattice alone is not accountable for the formation of cracks in the crystals of CBZ·2H_2_O. The strength and the temperature-dependence of electrostatic interactions, such as hydrogen bonds between CBZ and coformer, appear to influence the levels of stress to which the crystals are subjected that lead to fracture. Our findings show that the appropriate selection of coformer in multicomponent crystal forms, targetting superior mechanical properties, needs to account for the intrinsic stress generated by molecular vibrations and not solely by crystal anisotropy. Structural defects within the crystal lattice, although highly influenced by the crystallisation conditions and which are especially difficult to control in organic solids, may also affect breakage.

## Abstract 2

Crystal engineering has advanced the strategies of design and synthesis of organic solids with the main focus being on customising the properties of the materials.

## Graphical abstract

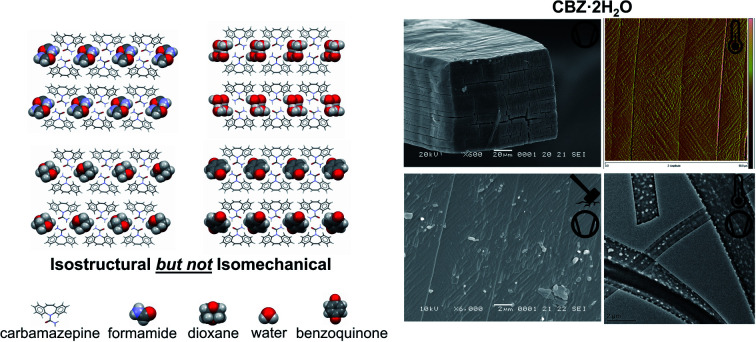

## 1 Introduction

Solid-state chemistry has long proven its importance in the field of pharmaceutical research for the development, manufacture and commercialisation of drug products.^1–5^ The recall cases of ritonavir, carbamazepine, hydrochlorothiazide/irbesartan, rotigotine and warfarin sodium 2-propanol solvate illustrate two major aspects that impact solid form selection during the development of medicines: (i) the solid form diversity and (ii) the particle/surface properties of the materials.^2,6–10^ While solid form diversity (*e.g.* polymorphism, hydrate/solvate formation) is extensively investigated, less attention is given to examining the possible variation in crystal surface characteristics and its impact on downstream processing.^11^ Indeed recent studies on the differences in surface structure at the nanoscale have proven informative about the polymorphic outcome of crystallisation,^12,13^ phase transitions,^14^ thermal^15^ and fluorescence^16^ properties. In the case of aspirin,^17^ for example, it has been shown that the well-known incompatibility of aspirin crystals with the excipient dicalcium phosphate dihydrate was related to the stronger interaction on one of the facets of aspirin surfaces.

The interplay between crystal form and surface, is therefore essential to the design, synthesis, and development of new solid materials with targeted chemical and physical properties.^4,18–20^ One such aspect of increasing interest involves the mechanical behaviour of organic solids, in particular the ability of a crystal to bend, move, break or heal.^21–29^ These characteristics play a significant role in the secondary manufacturing of Active Pharmaceutical Ingredients (APIs) such as milling and tableting of pure materials, and blending with excipients.^30–33^ At the standpoint of the influence of crystal structure, mechanical properties have been consistently explored on the basis of the anisotropic nature of a crystal and are known to be lattice, habit and surface specific.^34–43^ As of the examples studied thus far, it appears that the degree of mobility required in plastic and/or elastic deformations of organic solids is often affected by a balance of π–π stacking interactions, an absence of cross-linkage between molecular layers, and slip movements.^24,28,29,35,38^

**Fig. 1:**
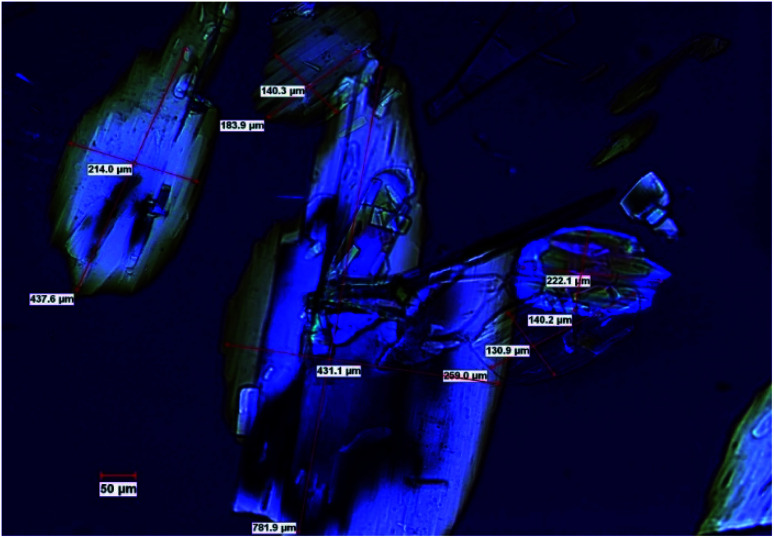
Optical micrographs of the CBZ·2H_2_O crystals prepared in a 9 kg scale batch, showing evidence of particle breakage and chipping (polarized light). The boxes show the dimensions of selected crystals ranging from 130 μm to 780 μm.

**Fig. 2:**
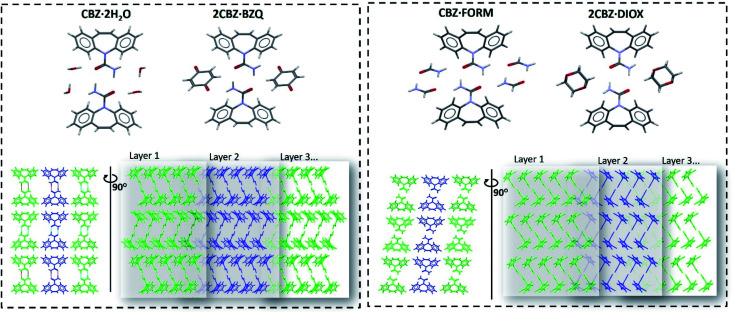
Molecular arrangement of the carbamazepine multicomponent materials showing their structural similarities (the respective coformer molecules are omitted in the bottom row of figures to facilitate comparison of the packing). Crystallographic data is available in Table S1.†

In this work we present a study of the breakage tendency of carbamazepine dihydrate (CBZ·2H_2_O) crystals and provide a possible explanation as to why the crystals have relatively poor mechanical properties and readily crumble into fine debris. This was previously observed in a study that involved the small-scale crystallisation of CBZ·2H_2_O.^44^ Also, Fig. 1 shows the product of a large-scale preparation of the dihydrate which resulted in substantial particle breakage and surface defects. The work was then expanded to the systematic assessment of the breakage of the dihydrate crystals from a process engineering perspective^45^ and, in this manuscript, is taken into a crystal engineering perspective. In particular, the current investigation presents a comparison of CBZ·2H_2_O with the cocrystal of CBZ with 1,4-benzoquinone (2CBZ·BZQ) and with the solvates of CBZ with formamide and 1,4-dioxane (CBZ·FORM and 2CBZ·DIOX, respectively). All these crystal forms are structurally related and present similar low index crystallographic planes of high atomic density (Fig. 2). At first, we expected these would all correspond to operative cleavage planes and we predicted similar mechanical features in the dihydrate and the other multicomponent crystal forms (as in isomechanical groups of similar strength of interaction and dimensions, a concept recently applied to organic solids by Gabriele *et al.*^42^). The results, however, do not support this hypothesis as fracture was seen only in the dihydrate. We therefore discuss the factors that contribute to the superior mechanical properties of 2CBZ·BZQ, CBZ·FORM and 2CBZ·DIOX in relation to CBZ·2H_2_O.§§For simplification purposes, both planes will be respectively called (020) and (100), although the correct crystallographic notation is different in CBZ·FORM and 2CBZ·DIOX. See Schneider-Rauber *et al*. (2020) for clarification.^44^

## 2 Results and discussion

### 2.1 Assessing the mechanical properties of carbamazepine dihydrate crystals

#### 2.1.1 Mechanical impact tests

Breakage experiments were performed on fresh CBZ·2H_2_O samples (see experimental for growth conditions) to evaluate the intrinsic mechanical properties of the dihydrate (Fig. S1–S3†). In these studies, the particles were accelerated towards a target to facilitate breakage.^46–48^ Higher impact velocities resulted in considerable change of crystal size with breakage. From Fig. S1† it is evident that the samples cleaved predominantly on {0*k*0} and {00*l*}, resulting in crystals with reduced dimensions along the *b* and *c* crystallographic directions. As a result, new {0*k*0} and {00*l*} surfaces were generated with new {00*l*} surfaces, in general, rougher and more irregular than the new {0*k*0} surfaces.

#### 2.1.2 The effect of vacuum

**Fig. 3:**
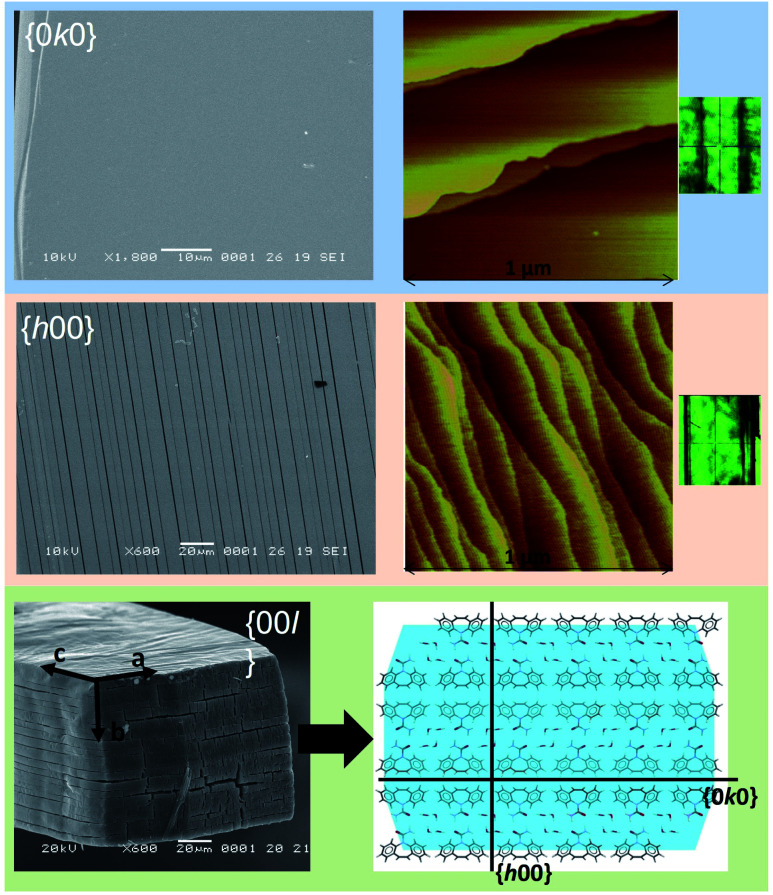
SEM and AFM (taken from Schneider-Rauber *et al.* (2020)^44^ with permission) micrographs representative of the {*h*00}, {0*k*0} and {00*l*} surfaces of CBZ·2H_2_O crystals (the optical micrograph on the right shows the crystal orientation during AFM). A model of the molecular arrangement of the {00*l*} surfaces with the illustration of {*h*00} and {0*k*0} within the packing is also presented.

The breakage of CBZ·2H_2_O crystals with exposure to vacuum was observed initially during sample preparation for Scanning Electron Microscopy (SEM)^44,49^ analysis. Freshly prepared CBZ·2H_2_O after exposure to vacuum revealed extensive cracking of the crystals on {*h*00} and {00*l*}, while none were observed on the {0*k*0} surfaces (Fig. 3). In contrast fresh crystals examined by Atomic Force Microscopy (AFM) confirmed the absence of cracking prior to vacuum and that they were artefacts of sample preparation.

**Fig. 4:**
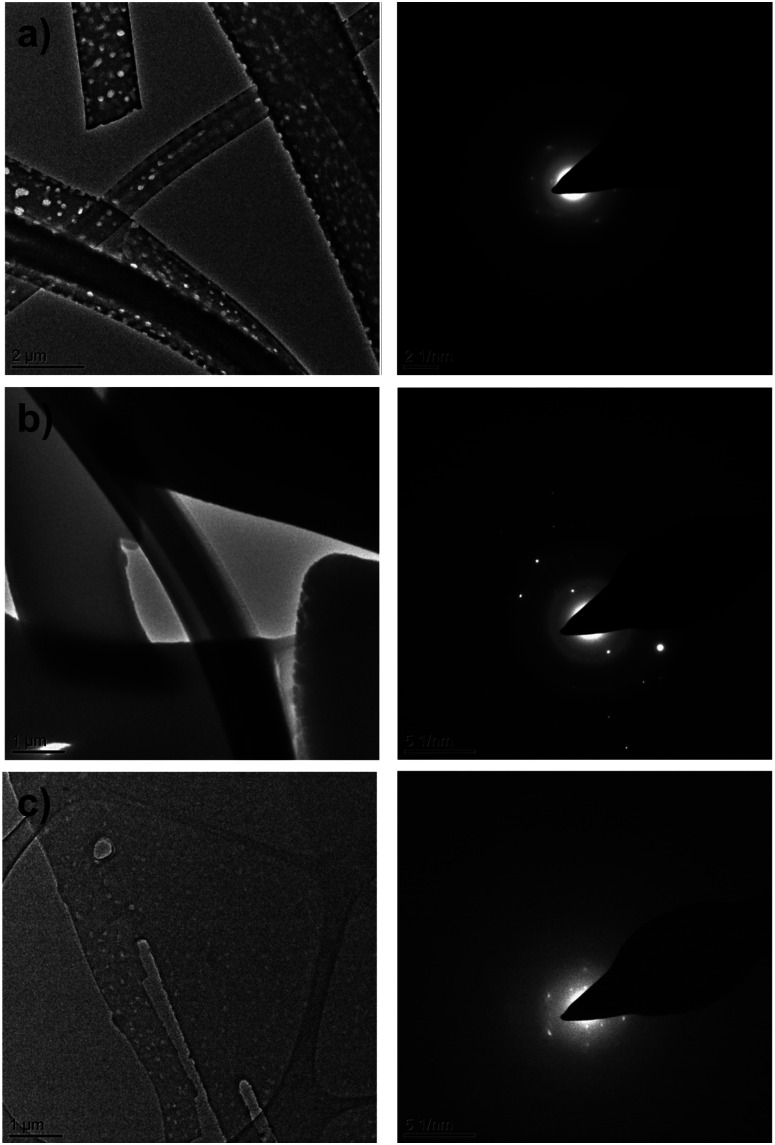
TEM bright field images (left) and electron diffraction patterns (right) of CBZ·2H_2_O crystals collected at room temperature (a), at cryogenic conditions (b) and at room temperature after warming from cryogenic conditions (c).

The destructive effect of vacuum on CBZ·2H_2_O crystals was also seen in room temperature Transmission Electron Microscopy (TEM). Bright field images (Fig. 4a) show that as the time of exposure to the microscope vacuum increased, the dihydrate crystals developed pores occasionally passing through the entire crystal. Reliable indexing of the associated diffraction patterns was, however, not possible, primarily because of limited electron beam exposure requirements.

Contrasting with these observations, when CBZ·2H_2_O crystals were examined in the TEM at lower temperatures (*ca.* 170 K) Fig. 4b bright field images showed the absence of pores over long periods of exposure to the vacuum. Additionally, the quality of the diffraction patterns collected improved considerably. As the sample temperature was increased, the crystals developed pores at temperatures above 0 °C, similar to the pores observed in the TEM experiments at room temperature (Fig. 4c). The quality of the diffraction patterns also decreased and clearly resembled the patterns shown in Fig. 4a. Furthermore, the analyses of anhydrous CBZ form II (Fig. S4 and S5†) show that the pores observed in the crystals of CBZ·2H_2_O are intrinsically related to CBZ dihydrate and its dehydration process under vacuum. Although cracks were not observed in the TEM of CBZ·2H_2_O, the effect of temperature on the damage observed in the vacuum environment of the TEM drew our attention to the contribution of intrinsic molecular movements, especially CBZ : water interactions, to the activation of cleavage. Ultimately, these characteristics may influence the general mechanical properties of CBZ·2H_2_O crystals.

#### 2.1.3 Effect of temperature at 0% RH

**Fig. 5:**
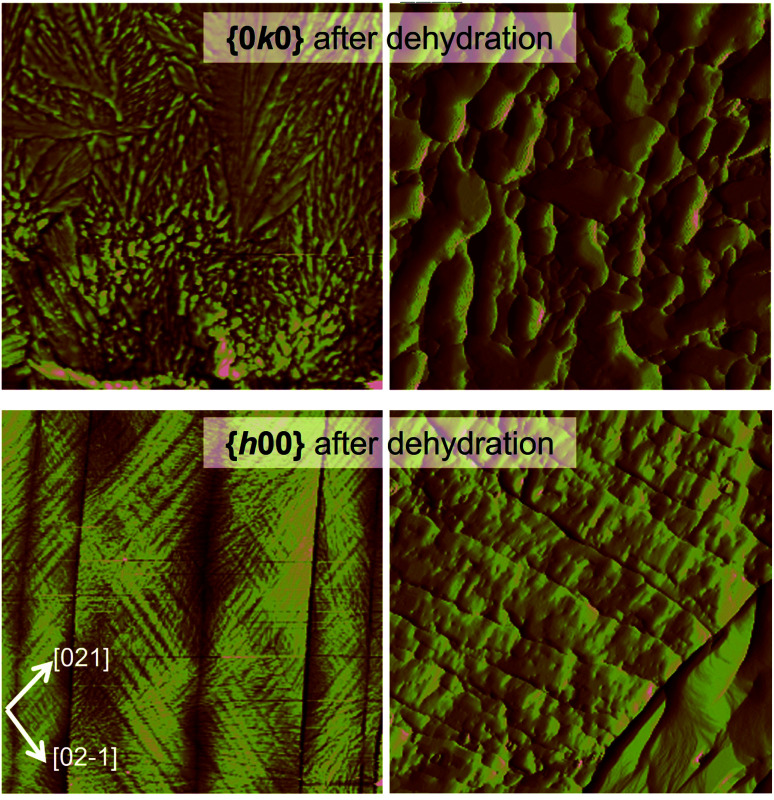
Topography of CBZ·2H_2_O crystal surfaces dehydrated at 40 °C/0% RH. {0*k*0}: scan of 30 μm and 5 μm, height and amplitude AFM images, left and right respectively. {*h*00}: scan of 60 μm and 5 μm, height and amplitude AFM images, left and right respectively.

The effect of temperature alone on the breakage of dihydrate crystal was also investigated. AFM analyses of CBZ·2H_2_O samples deliberately dehydrated at 40 °C in 0% RH show the formation of fractures on {*h*00}, the disruption of the steps observed on fresh {*h*00} and {0*k*0} surfaces and the formation of texture/striations characterised by small domains (Fig. 5). While {*h*00} faces showed a pattern of intersecting texture along the [021] and the [02−1] directions, {0*k*0} surfaces showed acicular domains of likely dehydrated crystals oriented along the needle axis.

The textured domains formed on {*h*00} surfaces (Fig. 5, S6 and S7†) appear continuous across surfaces separated by a crack. The orientation of the texture differs and results in domains related by 78.2 ± 3.3° (β in the figure) and is, in turn, related to the main cleavage direction by 36.1 ± 2.5° (α in the figure). It is noteworthy that a similar herringbone texture was also seen on {*h*00} surfaces of the CBZ·2H_2_O crystals subjected to impact and fresh crystals solely analysed by SEM. The effect is, however, more evident in thermally dehydrated samples.

Additional information on the cleavage of the CBZ·2H_2_O crystals is evident in the characteristics of the fractures. A few of the cracks seen on the {*h*00} surfaces, for instance, do not propagate the whole length of the crystal (Fig. S6 and S7†). Interestingly, this phenomenon was frequently seen as pairs of non-propagated cracks which developed to (or from) different crystal extremities. In summary, these findings, show that the cracks are generated to release the various types of local stress and strain formed throughout the crystal either because of dehydration or as a result of the release of mechanical strain.

### 2.2 Crystal structure analysis as a tool to understand the breakage tendency of carbamazepine dihydrate crystals

Crystal structure analysis explains the existence of two types of crystallographically oriented fractures for CBZ·2H_2_O as corresponding to (020) and (100) planes (Fig. 2 and 3). The {00*l*} surfaces show both types of fractures, while the cracks visible on the {*h*00} surfaces are solely related to (020).

**Fig. 6:**
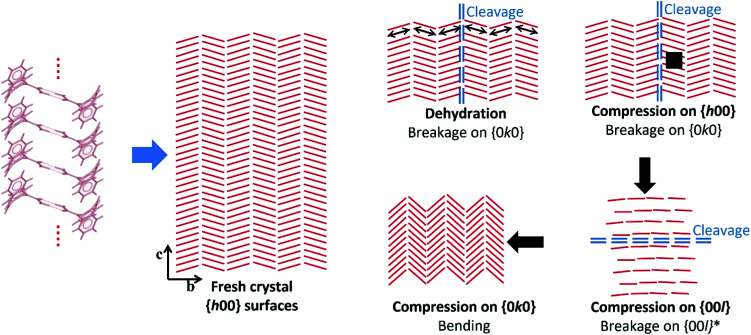
Scheme illustrating the hypothetical effects of dehydration and compression on the crystals of CBZ·2H_2_O based on the experimental data and the structural analyses. Bending upon compression on {0*k*0} is hypothesised after Reddy *et al.* (2005)^35^ and Reddy, Padmanabhan & Desiraju (2006).^38^ The red lines correspond to the CBZ dimers projected on the {*h*00} surfaces, and the arrows show the direction of the compressive forces.

In a previous report we showed that while the {*h*00} surfaces are composed of layers spaced by approximately 7.5 Å along the needle axis, {0*k*0} were composed of steps perpendicular to the needle axis with terraces of varied size demonstrating that the crystals are composed of superimposed {0*k*0} layers.^44^ In the earlier work, the relationship between the size of the {*h*00} surfaces and the number of {0*k*0} layers (*i.e.* thickness on the *b* direction) was associated with the existence of different particle trachts (*i.e.* variation of morphology as a result of the extent of development of faces).^44^ Here we show that these (0*k*0) layers also explain the mechanical properties of the dihydrate crystals since they represent the main cleavage planes within the structure. In fact, literature has shown that interactions between molecular layers formed by π–π stacking and their delamination phenomena may account for the direction of specific deformation in organic solids.^29,35,38^ The stacking and the molecular arrangement in the CBZ·2H_2_O crystal are translated into the different characteristics of {00*l*}, {*h*00} and {0*k*0} surfaces (Fig. 6).

It has been previously documented that water molecules may facilitate shear between interlocking layers in the crystal by a mechanism like lubrication.^50,51^ In CBZ·2H_2_O, however, this is less likely to happen because it would imply that slip/fracture occurs across the HBs of the CBZ dimers. In terms of significance, the most important type of fracture on CBZ·2H_2_O are the (020) planes as the fractures tend to run the entire crystal length. The (100) fracture planes have shown to be secondary because they propagate only in constrained regions under high stress such as between (020) cracks. The comparison of the attachment energies of both planes of the dihydrate shows that, indeed, the (020) plane presents the lowest attachment energy and thus corresponds to the most important cleavage plane in the structure (see Table 1).

**Table 1:** Attachment energies calculated for the carbamazepine multicomponent structures (taken from Schneider-Rauber *et al.* (2020), ref. 44)

Crystal form	Attachment energies^a^^,^^b^		
	*hkl*	*d*_*hkl*_ (Å)	*E*_att_ total (kJ mol^−1^)
2CBZ·BZQ	{0 2 0}	13.81	−20.68
	{1 0 0}	10.10	−43.91
CBZ·2H_2_O	{0 2 0}	14.36	−25.91
	{1 0 0}	9.79	−46.09
CBZ·FORM^c^	{0 1 1}	13.46	−28.19
	{0 1 −1}	10.11	−53.27

a The attachment energies of the 2CBZ·DIOX structure are not included (due to the disorder of the dioxane molecule).

b These values correspond to the lowest absolute attachment energies for the structures, except in the case of CBZ·FORM. In the case of CBZ·FORM, other planes show intermediate attachment energies in comparison to the planes shown on the table: (001) with −37.02 kJ mol^−1^, and (010) with −39.50 kJ mol^−1^. The results are not highlighted here, but they show differences in the predicted morphology from BFDH and the attachment energy method.

c The (011) and the (01−1) planes correspond to the (020) and (100) planes, respectively, in the other crystal forms.

The strain between (020) fractures is also manifested as herringbone striations visible on the {*h*00} surfaces. Such anisotropic surface features may indicate the orientation of the strain direction and the movement history of the cracks on the {*h*00} faces, especially the shear stress that was present before fracture occurred.^52^Fig. 6 and S6† show the corresponding structural features which are believed to be correlated to these striations: the dimers of CBZ and the (020) cleavage planes. Kachrimanis & Griesser^53^ observed intersecting cracks upon thermal dehydration of CBZ·2H_2_O. The fractures were characterised by equivalent angles to those reported here (*i.e.* 40.6 ± 1.2° in relation to the needle axis and crossing at 81.5 ± 1.6°). The authors reported, however, the formation of these features on {0*k*0} surfaces as opposed to {*h*00} surfaces, as shown here.

Cracks parallel to the needle axis and which correspond to the (020) crystallographic planes have also been reported in the literature. Khoo *et al.*^49^ attributed the formation of these cracks on {*h*00} to the effect of early stages of dehydration. In our work, we suggest that the cause of fractures may be either dehydration (*e.g.* caused by vacuum and/or thermal treatment) or purely the release of mechanical strain (*e.g.* as in the effect of impact).

While the texture apparent on {*h*00} surfaces and the cleavage on (020) are clearly correlated to crystal structure, the breakage perpendicular to the needle axis seen in the impact tests does not appear to be related to any weak crystal plane. The high stress exerted on the crystals during impact may modify the lattice in comparison to the perfectly homogeneous and flawless non-stressed crystal, as shown in Fig. 6. In particular, we notice that the large bending force caused by the high aspect ratio of the dihydrate particles may influence the breakage perpendicular to the needle axis.

### 2.3 Improved mechanical properties of BZQ, FORM and DIOX crystals compared to carbamazepine dihydrate

**Fig. 7:**
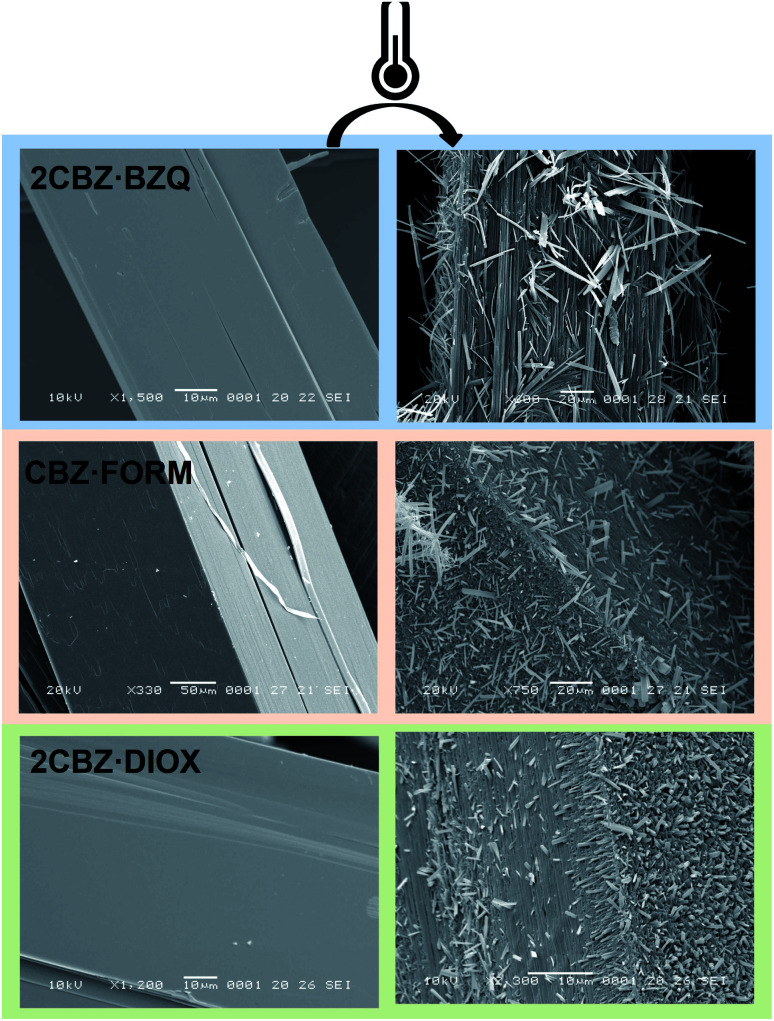
SEM micrographs of fresh and thermally treated crystals of carbamazepine multicomponent crystals. From top to bottom, 2CBZ·BZQ after heating to 150 °C, CBZ·FORM after heating to 170 °C, and 2CBZ·DIOX after heating to 120 °C (N_2_, heating rate of 10 °C min^−1^). The formation of whiskers and needles on the surface of the particles is characteristic of the formation of CBZ polymorph I (*P*1̄). For more information on the polymorphism and surface diversity arising from stress-induced transformations of CBZ multicomponent crystal forms, (see ref. 55).

The analysis of the structural properties of CBZ·2H_2_O crystals show that they are prone to breakage as a result of applied external forces and also as a result of internal stresses generated during dehydration. The brittleness of the material is surface-specific as different surfaces show different susceptibilities to crack formation. It was shown in Section 2.2 that this characteristic relates to the crystal topography and the structure of CBZ·2H_2_O. However, the study of the responses of 2CBZ·BZQ, CBZ·FORM and 2CBZ·DIOX to the stress generated by vacuum and thermal treatment (Fig. 7) shows that, although these materials are structurally related, only CBZ·2H_2_O appears to consistently develop fractures and visible herringbone texture on the {*h*00} surface. For instance, conventional TEM analyses of 2CBZ·BZQ (Fig. S9†), *i.e.* without cryogenic protection, show no cracks or pores during examination and diffraction patterns of high crystallinity.

Examination of the arrangement of CBZ molecules in CBZ·2H_2_O, 2CBZ·BZQ, CBZ·FORM and 2CBZ·DIOX (Fig. 2) suggests that the crystallographic planes which appear to involve the least energy for molecular movement (*i.e.* weakly bound/cleavage planes) are nominally the same. They are the planes along the dibenzoazepine groups (*i.e.* the (020) plane), and the planes which are longitudinal to the CBZ dimers and cross the channels formed by the guest molecules (*i.e.* the (100) plane). A comparison of the attachment energies within the crystal forms (Table 1) shows that they are equivalent and does not explain the difference in the mechanical properties.

The texture seen on the {*h*00} surfaces of the dihydrate, however, gives a hint to the phenomena explaining its behaviour. These surface features show that the stress associated with cleavage runs parallel to the CBZ dimers. It indicates that these supramolecular structures may be related to the generation and the transfer of stress to (020). Considering the CBZ dimers as tensile axes acting on the (020) plane, the larger the angle between dimer and plane, the stronger the force component acting normal to (020) and contributing to fracture activation. In contrast to cleavage, the component which runs parallel to (020) accounts for slipping. Frictional sliding is caused by edge dislocation movement that does not damage the crystal but is expected to slip through the crystal and ‘heal’. Our examination reveals that the angles between the dimers and the crystal planes, *i.e.* geometric parameters, are different in CBZ·2H_2_O, 2CBZ·BZQ, CBZ·FORM and 2CBZ·DIOX (Table 2). The analysis show that the cleavage component in CBZ·2H_2_O may be more important than in 2CBZ·BZQ, CBZ·FORM and 2CBZ·DIOX. This is also illustrated in weak intermolecular interactions between CBZ molecules across (020) and strong π–π interactions in the dihydrate (Table 2).^29^

**Table 2:** Comparison of carbamazepine multicomponent crystal forms according to intermolecular interactions, the angle between CBZ dimers and the (020) plane, and the enthalpy of vaporisation of the guest molecules

Crystal form	Intermolecular energy^a^ (kJ mol^−1^)				Dimer ∢ (020) plane (°)	Vaporisation enthalpy^b^ (kJ mol^−1^)
	CBZ + guest	CBZ dimer	CBZ stack	CBZ across (020)		
CBZ·2H_2_O^c^	−29.9	−37.5	−50.1	−20.2	67.4	149.4
	−13.2					
2CBZ·BZQ	−22.6	−33.1	−46.4	−21.2	61.2	100.2
CBZ·FORM^d^	−18.9	−31.7	−44.3	−21.5	59.8	92.8
	−21.9					
	−23.1	−34.3	−44.2			
	−21.3					
2CBZ·DIOX^e^	−27.5	−30.0	−48.2	−21.0	63.2	—

a Obtained from Mercury® (UNI Intermolecular Potentials) and previously reported elsewhere (see ref. 55).

b Calculated vaporization enthalpies per formula unit for the decomposition of multicomponent materials into CBZ polymorph (I). The attachment energies of the 2CBZ·DIOX structure are not included (due to the disorder of the dioxane molecule).

c CBZ is linked to molecules of water through O–H⋯O hydrogen bonds between the hydroxyl group of water and the carbonyl of CBZ, but also *via* weaker N–H⋯O hydrogen bonds between the amine of CBZ and the water oxygen.

d The reason why four types of CBZ : guest interactions as well as two types of CBZ stacking and two dimers exist in CBZ·FORM is because its asymmetric unit consists of two CBZ and two formamide molecules in a triclinic cell. The non-equivalent CBZ molecules form different dimers which, in turn, interact with non-equivalent formamide molecules.

e No calculations of vaporisation enthalpy were performed for 2CBZ·DIOX due to complications related to the disorder of the solvent molecule. The intermolecular energy were calculated from the doubled unit cell (see ref. 44).

In combination with the decreased ability to accumulate strain, CBZ·2H_2_O crystals may also be subjected to higher levels of stress. For instance, strong intermolecular interactions between CBZ and water indicate a substantial level of stress being produced because of the molecular movements which occur during dehydration. This effect is illustrated in the periodic density functional theory (DFT) calculation of the coformer release (Table 2), as the dihydrate has shown the highest energy release from guest evolution. The onset temperature of desolvation and sublimation, however, does not appear to play a significant role in the breakage tendency.^54^

As a summary, the discussion above suggests that CBZ·2H_2_O is particularly prone to the formation of cracks because of a combination of (i) the orientation of the CBZ dimers with respect to (020), and (ii) the strength of interactions between CBZ and water, between CBZ molecules across the cleavage plane and CBZ molecules related by stacking. Hydrogen bonds are important to the mechanical properties, especially the fracture condition, because they operate at long distances and act as stress axes orienting the molecular movements within the crystal. This characteristic drives the mechanisms by which molecular fluctuations/vibrations originating from phenomena which are external (*e.g.* impact and compression)^55,56^ and/or internal (*e.g.* physicochemical reactions and thermal motion)^21,57^ to the crystal may lead to cleavage or motion.

It is likely that the periodicity of the cracks on the dihydrate surface, indicates a critical value of stress which can be tolerated by the lattice before fracture occurs. Once the stress exceeds this value, a fracture propagates on (020). Considering the crystal structure and the topography of CBZ·2H_2_O, we propose that stress starts building up as the number of molecular layers increase on *b*. In comparison, the crystal structures of the other CBZ multicomponent materials may tolerate a larger amount of critical stress because of the smaller magnitude of their intermolecular interactions, and because of the orientation of the stress axes (*i.e.* CBZ dimers) relative to the cleavage plane.

### 2.4 A role for defects?

A further aspect that may influence the mechanical properties of CBZ·2H_2_O, but is challenging to assess, is the presence of crystal defects. Harris *et al.*,^58^ have reported the existence of twinning of the (100) plane in CBZ dihydrate crystals. The authors suggested that the twinning could be of a domain-type disorder (with domain sizes of tens to hundreds of Å) or could occur on a microscopic level (multiple micro-twinning or penetration twinning).

In the present work, defects on the *b* axis were evidenced in one electron diffraction pattern of thin CBZ·2H_2_O crystals analysed by TEM (Fig. S10†). We have also seen the formation of striated domains in different directions on the {*h*00} surfaces of larger crystals. Both observations show experimental evidence of boundaries and domain dimensions in SEM images of fresh crystals, and in AFM and SEM images of crystals after dehydration. In addition to that, the TEM images and the streaking observed in several electron diffraction patterns of 2CBZ·BZQ may also be suggestive of stacking faults, thin ordered domains or finely twined structures (Fig. S9†).

For the time being, the striated domains remain intriguing, if not fully understood. One remaining question, therefore, is how defects affect the mechanical properties of CBZ·2H_2_O (and other) crystals. Twinning could potentially modify the strain in the lattice and the local intensity of the tensile stress acting on the (020) planes, but the presence of grain boundaries could also affect crack propagation by absorbing energy associated with stress. It is noteworthy, however, that the size of the domains is a sample-specific property which is highly influenced by the crystallisation conditions and especially difficult to control in organic solids.^59^

## 3 Conclusions

The case of CBZ·2H_2_O illustrates the effect of crystal anisotropy on the structural properties of organic solids resulting from mechanically-induced stress and stress resulting from dehydration. For instance, because of molecular arrangement, the {*h*00} surfaces show brittle behaviour while the {0*k*0} surfaces tend to be more plastic with respect to applied stresses which can be either external (*e.g.* mechanical mixing during the crystallisation process) or internal (*e.g.* as a consequence of dehydration) of the dihydrate crystals. A comparison of CBZ·2H_2_O with 2CBZ·BZQ, CBZ·FORM and 2CBZ·DIOX, however, has shown that the source and the magnitude of tensile stresses acting on the’ weak planes of the crystals must also be considered in determining the likelihood of breakage. This is part of the explanation as to why the dihydrate is more prone to the formation of cracks, although it is structurally similar to the other crystal forms. In terms of structure, the critical stress which can be tolerated by the CBZ lattice before cleavage depends on the orientation of the CBZ dimer axes relative to the cleavage plane. The level of stress to which the crystal is intrinsically subjected, in turn, depends on the dependence of molecular vibrations with temperature and the strength of interaction between CBZ molecules and CBZ : coformer pairs. Both aspects should be considered when attempting to design multicomponent crystal forms with appropriate structural (*i.e.* mechanical) properties, especially labile pharmaceutical materials and dynamic crystals aimed at various innovative technological applications. Although previous work has already addressed mechanical anisotropy in organic crystals with respect to thermal expansion and H-bonding patterns,^35,38,39,60–63^ our discussion shows that molecular vibrations and defects may add to the scope of future investigations.

## 4 Experimental

### 4.1 Materials

Solvents were supplied by Sigma–Aldrich Company Ltd and used without further purification. Carbamazepine and 1,4-benzoquinone were obtained from Alfa Aesar and Thermo Fisher Scientific, respectively.

### 4.2 Sample preparation

The crystallisation conditions were selected based on previous studies (Fig. S8†).^44^ The CBZ·2H_2_O, 2CBZ·BZQ, CBZ·FORM and 2CBZ·DIOX samples were prepared from cooling (−5 °C h^−1^) ethanolic solutions containing CBZ and the respective coformer.

The nucleation was spontaneous and, to avoid particle breakage, no agitation was used. The crystals were harvested by vacuum filtration and dried under room conditions. In general, the samples were prepared in small batches yielding approximately 0.5–1.0 g.

CBZ·2H_2_O sample used in the impact tests was prepared in a batch yielding approximately 500 g. The crystals presented {*h*00} dominant surfaces.^44^ A crystallisation reactor of 10 L was used and the process involved the combination of forward anti-solvent addition, wet milling of “dry” seeds (Ultra-Turrax® dispersers – IKA-T10) and heat and cool cycles (low supersaturation and final ethanol : water proportion of 20 : 80, v/v).

A 9 kg batch (Fig. 1) was prepared using a crystallisation reactor of 170 L and the method involved the forward addition of water into ethanolic solution of CBZ and the *in situ* seed generation (Ultra-Turrax® dispersers – IKA-T25).

### 4.3 Powder X-ray diffraction (PXRD)

PXRD was used to confirm the crystal phase identity of the materials prepared (Fig. S11–S14†). Additionally, the diffractograms were used to assess the sample's dominant crystal faces which were inferred by preferred orientation. The measurements were performed at room temperature on a PanAlytical X'Pert PRO Multi-Purpose Diffractometer using Cu Kα radiation (*λ* = 1.5418 Å) and an X'Celerator detector. The X-ray generator was set at a voltage of 40 kV and current of 40 mA. Samples were placed onto a flat glass slide and scanned from 3° to 40° 2*θ* using a total scanning time of 5 minutes.

### 4.4 Surface analyses

Scanning electron micrographs (SEM) were taken using a JEOL JSM-5510LV scanning electron microscope. The samples were mounted with adhesive conducting tape over an aluminium holder and sputtered with gold for 3 minutes (Agar Sputter Coater). The vacuum within the sputter unit was *ca.* 1 × 10^−3^ torr.

AFM images were recorded using a MultiMode atomic force microscope (NanoScope IIIa controller; Veeco). The stage was equipped with a video microscope to position the sample on the J scanner base. The samples were fixed to glass coverslips using sticky tabs over stainless steel sample holders. Before AFM analysis, the samples were observed on the metallic discs using a binocular GX reflective optical microscope equipped with a Motticam 2000 microscope digital camera. All images were recorded in tapping mode using TESP 15 series (HQ:NSC15/Al BS) sharpened silicon probes with nominal spring constant of 40 N m^−1^ and nominal resonance frequency of 325 kHz (μmasch). The scan rate was changed according to the size of the scan area and the features observed on the surface. The scans were analysed using NanoScope software version 6.13 (Veeco). Each height image was processed using the plane-fitting third-order and the flatten zero-order commands in the software. For the amplitude images, the plane-fitting zero-order command was performed.

### 4.5 Transmission electron microscopy (TEM)

Conventional TEM analyses were performed at room temperature on a FEI Tecnai TF20 instrument operating at 200 kV. Data were directly collected as digital images by a CCD camera Gatan Orius SC600A. The relative rotation between TEM images and electron diffraction patterns is 90°. The diffraction patterns had the position of the reflections and the angles between them measured in Image J software. Three *d*-spacing values per diffractogram and their respective angles were combined and matched to the reported structures to check the identity of the crystals. As a final step, Crystal Maker Single Crystal v1.3 was used to compare the experimental diffraction pattern to the simulated diffraction pattern of the indicated zone axis, which was obtained from the reported structures retrieved from CSD.

Samples were directly prepared onto lacey-carbon films supported on 300 mesh copper grids. The sample preparation of CBZ : 2H_2_O and CBZ polymorph II typically consisted of evaporating solutions of CBZ in ethanol : water and tetrahydrofuran (respectively) directly onto the TEM grid. The preparation of 2CBZ : BZQ samples typically consisted of evaporating acetonitrile solutions containing CBZ and BZQ directly onto the TEM grid. Specimens for cryoTEM analyses were prepared by plunge-freezing the TEM grids in liquid ethane. The samples were transferred in a cryo-holder under temperature control and were maintained at 170 K during the analyses. In certain cases, the temperature was allowed to increase in order to perform conventional TEM analyses using the same grid (no heating rate control).

### 4.6 Impact tests

Breakage experiments were performed with the Scirocco disperser of the Mastersizer 2000 and the dispersion unit of Morphologi G3, both of Malvern Panalytical, Malvern UK.^46–48^ Different dispersion pressures were tested for each equipment to vary the impact velocity. They were 0.1 and 1 barg in the Scirocco disperser, and 1 and 2 barg pressure pulses of 20 ms duration for Morphologi G3. According to calculations based on Computational Fluid Dynamics (CFD),^46–48^ it is estimated that the impact velocities are as follows: 20 and 30 m s^−1^ for Scirocco, and 2 and 2.5 m s^−1^ for Morphologi G3. A small quantity of crystals was fed manually and following the impact stage, they were collected for damage observations.

### 4.7 Use of the cambridge structural database (CSD)

Conquest was used to search for deposited crystal structures. Mercury was used for general structural analysis and visualisation as well as obtaining values of intermolecular energy calculated using the UNI force-field potentials.^64–66^ The refcodes of the crystal structures retrieved from the CSD are as follows: CBZ·2H_2_O (FEFNOT02),^58^ 2CBZ·BZQ (UNEYOB),^67^ CBZ·FORM (UNIBOI),^67^ and 2CBZ·DIOX (QABHOU).^44^ The structure of 2CBZ·BZQ is isostructural and directly comparable to that of CBZ·2H_2_O. The unit cell of CBZ·FORM is metrically comparable to that of 2CBZ·DIOX, but with a different cell setting. The unit cell of CBZ·FORM could be transformed to clarify the face indexing, but it was decided to retain the setting present for UNIBOI in the CSD (as in Table S1†).

### 4.8 Calculation of attachment energies

The attachment energy calculation was performed with the COMPASS force field using the Morphology module in Materials Studio 5.5 (Biovia). Prior to calculations, the crystal structures were geometry optimized.

### 4.9 Periodic DFT calculation of the enthalpy of coformer evaporation

Periodic DFT calculations were used to compute the energies of guest release from multicomponent forms of CBZ. The calculations were performed using a plane-wave DFT code CASTEP19.^68^ Experimental crystal structures of CBZ form I, CBZ·2H_2_O, 2CBZ·BZQ CBZ·FORM and 2CBZ·DIOX (supercell structure)^44^ were converted into CASTEP format using the program cif2cell.^69^ In order to account for the energetic effect of DIOX molecule disorder in the 2CBZ·DIOX crystal structure, two calculations were performed, each including one of the disorder configuration. The overall electronic energy of this structure was computed as an average of two configurations. The calculations were performed using a PBE^70^ functional combined with many-body dispersion (MBD*)^71–73^ correction. The plane-wave basis set was truncated at 800 eV cutoff, and the 1^st^ electronic Brillouin zone was sampled with a 2π × 0.05 Å^−1^ Monkhorst–Pack *k*-point spacing.^74^ The crystal structures were geometry-optimized with respect to atom coordinates and unit cell parameters, subject to the symmetry constraints of their respective space groups. In addition, the gas-phase energies of H_2_O, BZQ and FORM molecules were calculated by placing them in a cubic box with a fixed dimension of 30 Å, and performing geometry optimization using the same settings used for the crystal structures. The enthalpies of coformer evaporation of CBZ·2H_2_O, 2CBZ·BZQ CBZ·FORM and 2CBZ·DIOX were calculated by combining the lattice energies of the CBZ polymorph I to the energy of the guest molecule in the gas phase, and subtracting this value from the calculated lattice energies of the respective multicomponent form. Polymorph I was selected because previous studies have shown that the thermal treatment of CBZ·2H_2_O, 2CBZ·BZQ and CBZ·FORM result in CBZ Form I.^54^

## Abbreviations

AFM: Atomic force microscopy
CBZ: Carbamazepine
2CBZ·BZQ2:1: 2:1Carbamazepine cocrystal with 1,4-benzoquinone
2CBZ·DIOX2:1: 2:1Carbamazepine solvate with dioxane
1:1 CBZ·FORM: Carbamazepine solvate with formamide
CBZ·2H_2_O: Dihydrate of carbamazepine
CSD: Cambridge structural database
OM: Optical microscopy
PXRD: Powder X-ray diffraction
SEM: Scanning electron microscopy
TEM: Transmission electron microscopy

## Author contributions

All authors contributed to the development of the project and interpretation of results. GS-R, AYS and WJ to the writing of the manuscript.

## Funding

AbbVie (North Chicago, USA) sponsored and funded the study, contributed to the design, participated in the collection, analysis, and interpretation of data, and in writing, reviewing, and approval of the final publication. All AbbVie authors are employees of AbbVie and may own AbbVie stock.

## Conflicts of interest

The authors declare there were no conflicts of interest in this work.

## Supplementary Material

SC-012-D1SC03095G-s001
