# Supplementary material for: Understanding stress-induced disorder and breakage in organic crystals: beyond crystal structure anisotropy
Source: Chem Sci. 2021 Oct 20;12(42):14270–80. doi: 10.1039/d1sc03095g (PMC8565387; doi:10.1039/d1sc03095g)
Supplement: SC-012-D1SC03095G-s001 [file SC-012-D1SC03095G-s001.pdf]

Electronic Supplementary Material (ESI) for Chemical Science.
This journal is © The Royal Society of Chemistry 2021
Please do not adjust margins

ARTICLE – Supporting Information

Understanding stress-induced disorder and breakage in organic crystals: beyond crystal structure anisotropy

Gabriela Schneider-Rauber, Mihails Arhangelskis, Wei-Pin Goh, James Cattle, Nicole Hondow, Rik Drummond-Brydson, Mojtaba Ghadiri, Kushal Sinha, Raimundo Ho, Nandkishor K. Nere, Shailendra Bordawekar, Ahmad Y. Sheikh*, and William Jones*

Contents

Crystal data....................Page 1
SEM images of CBZ·2H_2O subjected to impact tests....................Page 2
Transmission electron microscopy (TEM) of CBZ Form II....................Page 5
Sample identification – Additional SEM images of CBZ·2H_2O....................Page 6
Transmission electron microscopy (TEM) of 2CBZ·BZQ....................Page 8
Transmission electron microscopy (TEM) of CBZ·2H_2O....................Page 9
Sample identification – PXRD....................Page 10
References....................Page 12

Crystal data

Table S1 - Crystallographic data of the CBZ multicomponent crystal forms

Crystal form	CBZ·2H_2O	2CBZ·BZQ	CBZ·FORM	2CBZ·DIOX[a]
Formula	$(C_{15}H_{12}N_2O)\ (H_2O)_2$	$(C_{15}H_{12}N_2O)_2\ (C_6H_4O_2)$	$(C_{15}H_{12}N_2O)\ (CH_3NO)$	$(C_{15}H_{12}N_2O)_2\ (C_4H_8O_2)$
MW/g.mol^{-1}	272.30	580.63	281.31	560.63
T/K	120	100	100	180
Crystal system	Monoclinic	Monoclinic	Triclinic	Monoclinic
Space group	$P2_1/c$	$P2_1/c$	P-1	$P2_1/n$
a/Å	10.066(2)	10.3335(18)	5.1077(11)	16.7776(16)
b/Å	28.719(5)	27.611(5)	16.057(3)	4.9175(5)
c/Å	4.831(1)	4.9960(9)	17.752(4)	17.991(17)
α/°	90	90	73.711(3)	90
β/°	103.45(1)	102.275(3)	89.350(3)	107.814(5)
γ/°	90	90	88.636(3)	90
V/Å^3	1358.3	1392.9	1397.1	1413.2(2)
Z/Z'	4/1	2/0.5	4/2	2/0.5
ρ/g.cm^{-3}	1.332	1.384	1.337	1.318
R-factor/%	7.29	5.45	7.66	7.27
CSD refcode	FEFNOT02	UNEYOB	UNIBOI	QABHOU
Reference	Harris *et al.* (2005)[1]	Fleischman *et al.* (2003)[2]	Fleischman *et al.* (2003)[2]	Schneider-Rauber *et al.* (2020)[3]

[a] The structure of 2CBZ·DIOX can be described with a supercell in space group P21/c, with dimensions a = 28.071(2), b = 4.9171(4), c = 20.5138(14) Å, α = γ = 90, β = 94.177(4)°. Details are given in Schneider-Rauber *et al.* (2021).

This journal is © The Royal Society of Chemistry 20xx

Please do not adjust margins

Please do not adjust margins

SEM images of CBZ·2H_2O subjected to impact tests

The Scirocco disperser of Mastersizer 2000 and the aerodynamic disperser of Morphologi G3 (Malvern Panalytical) were used for these studies.[4–6] Tests using these instruments provided distinct evidence for the extent of mechanically-induced breakage. At high impact velocities (**Figure S1**), crystal size changed considerably with breakage. At low particle impact velocity (**Figures S2** and **S3**), overall particle shape and size were generally maintained and breakage was limited to geometric fracture along the shortest dimension.[7]

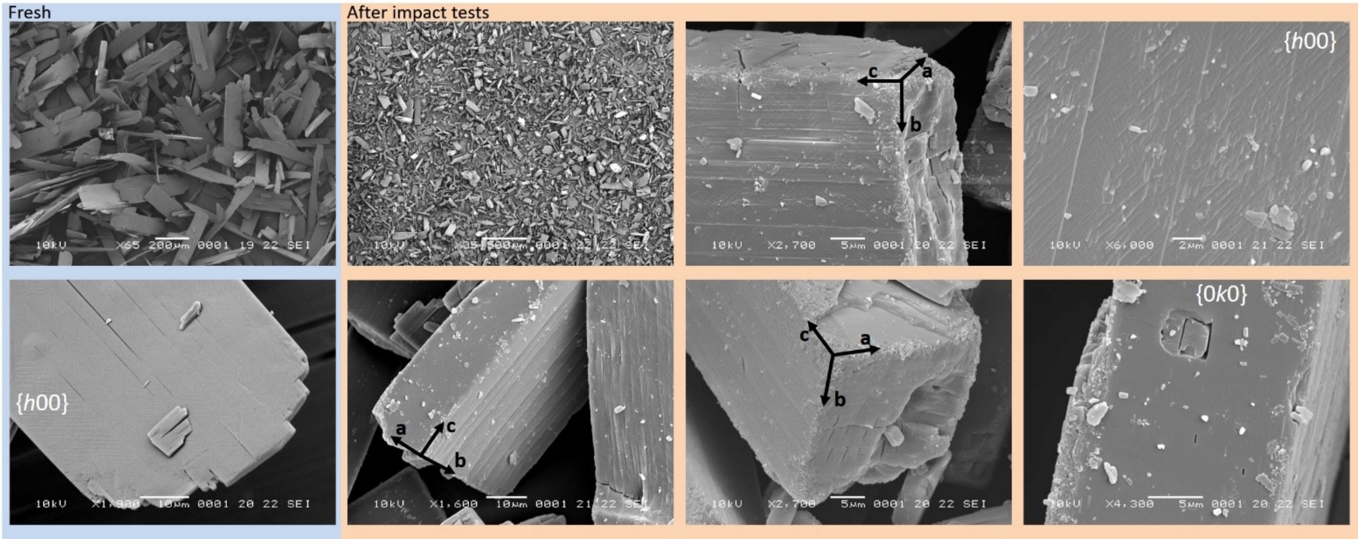

Figure S1 – SEM micrographs comparing (in blue) fresh CBZ·2H_2O crystals to (in pink) post impact samples (Scirocco disperser, different dispersion pressures giving rise to different impact velocities in the range 20-30 m/s). Face assignments are based on the effect of the vacuum on the appearance of the {*h*00} and {0*k*0} faces - see manuscript.

 This journal is © The Royal Society of Chemistry 20xx

Please do not adjust margins

Please do not adjust margins

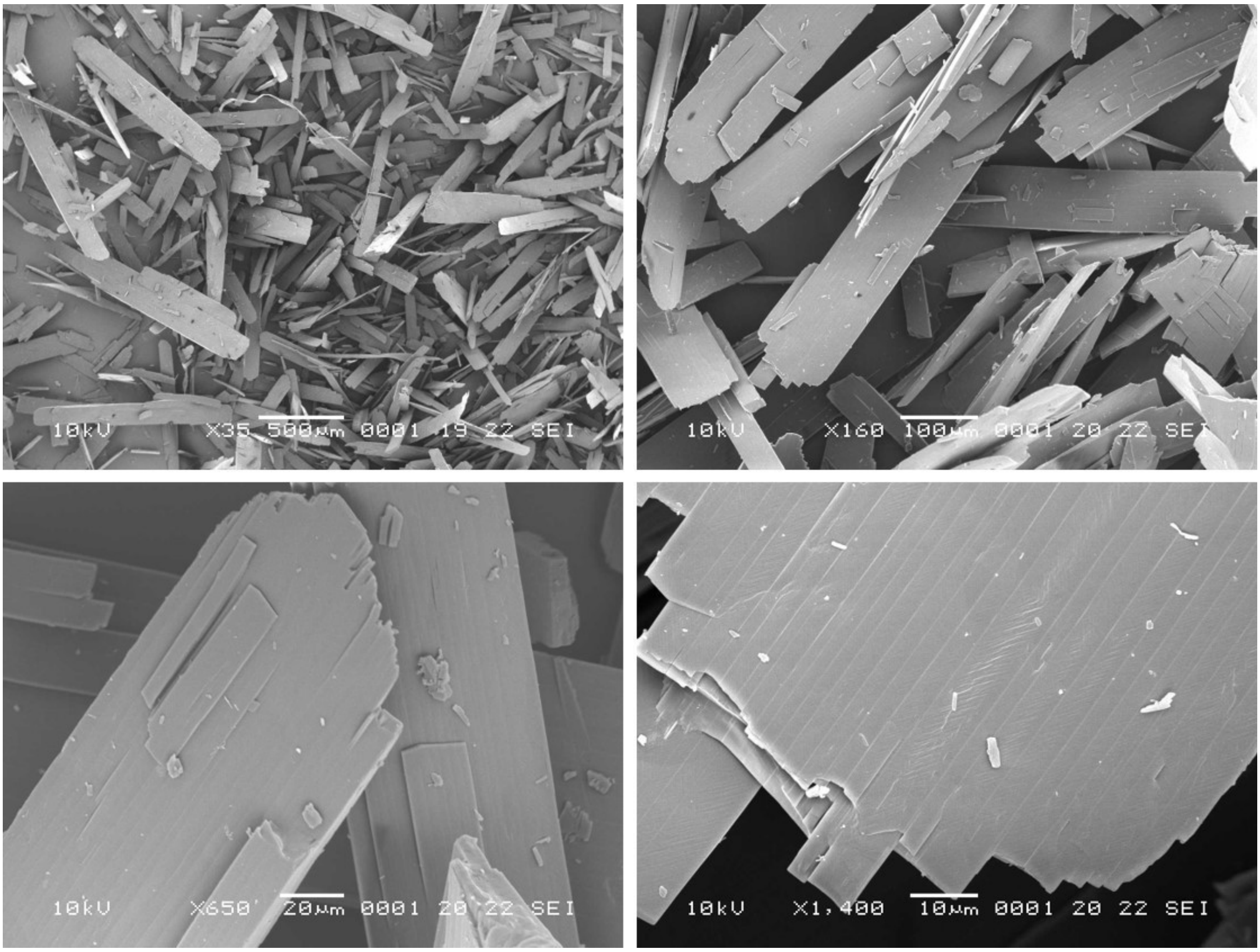

Figure S2 – SEM micrographs showing the results of impact tests at low impact velocities on the CBZ·2H_2O sample (Morphologi G3, 1 bar of dispersion pressure).

This journal is © The Royal Society of Chemistry 20xx

Please do not adjust margins

Please do not adjust margins

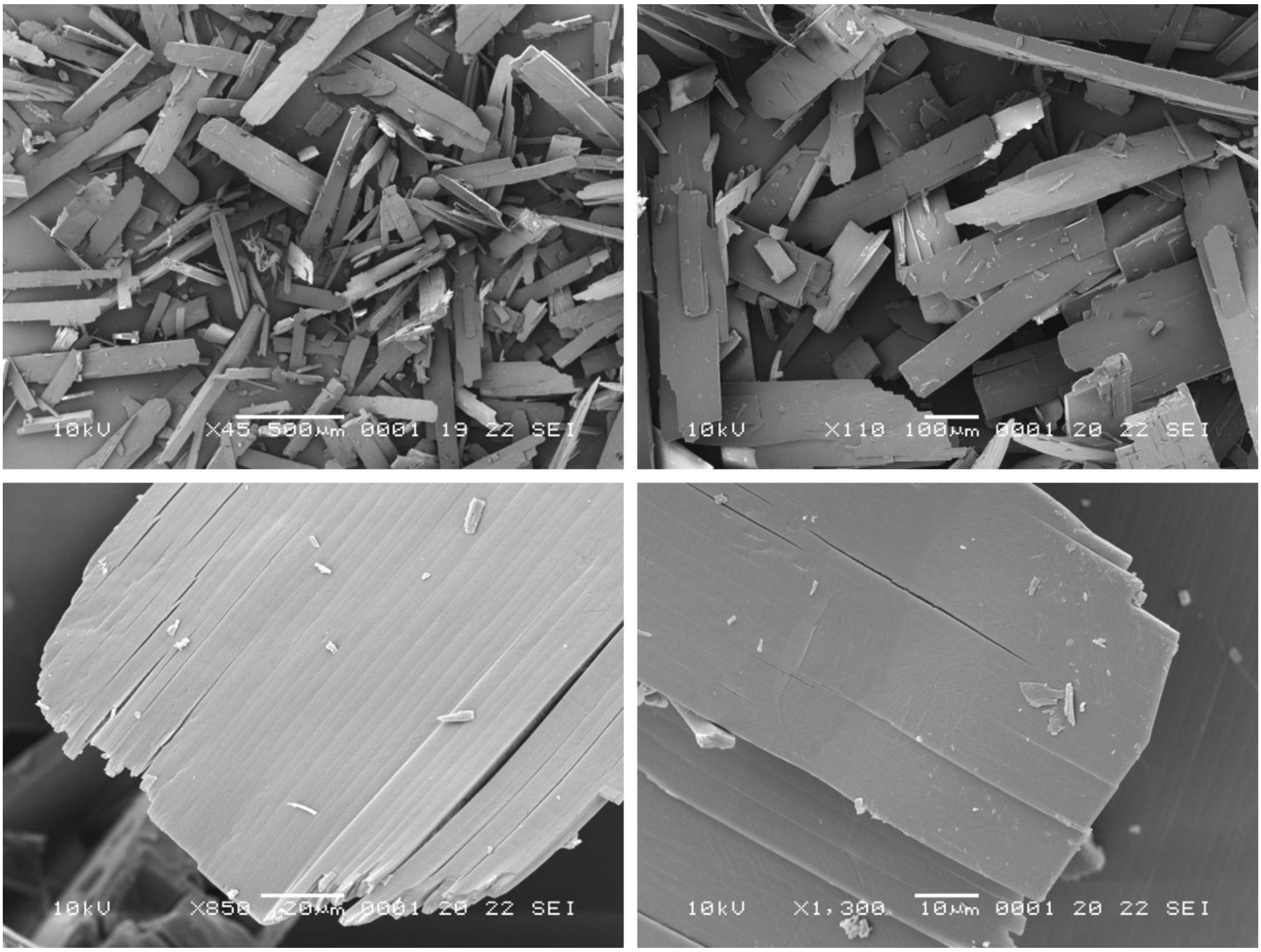

Figure S3 – SEM micrographs showing the results of impact tests at low impact velocities on the CBZ·2H_2O sample (Morphologi G3, 2 bar of dispersion pressure).

This journal is © The Royal Society of Chemistry 20xx

Please do not adjust margins

Please do not adjust margins

Transmission electron microscopy (TEM) of CBZ Form II

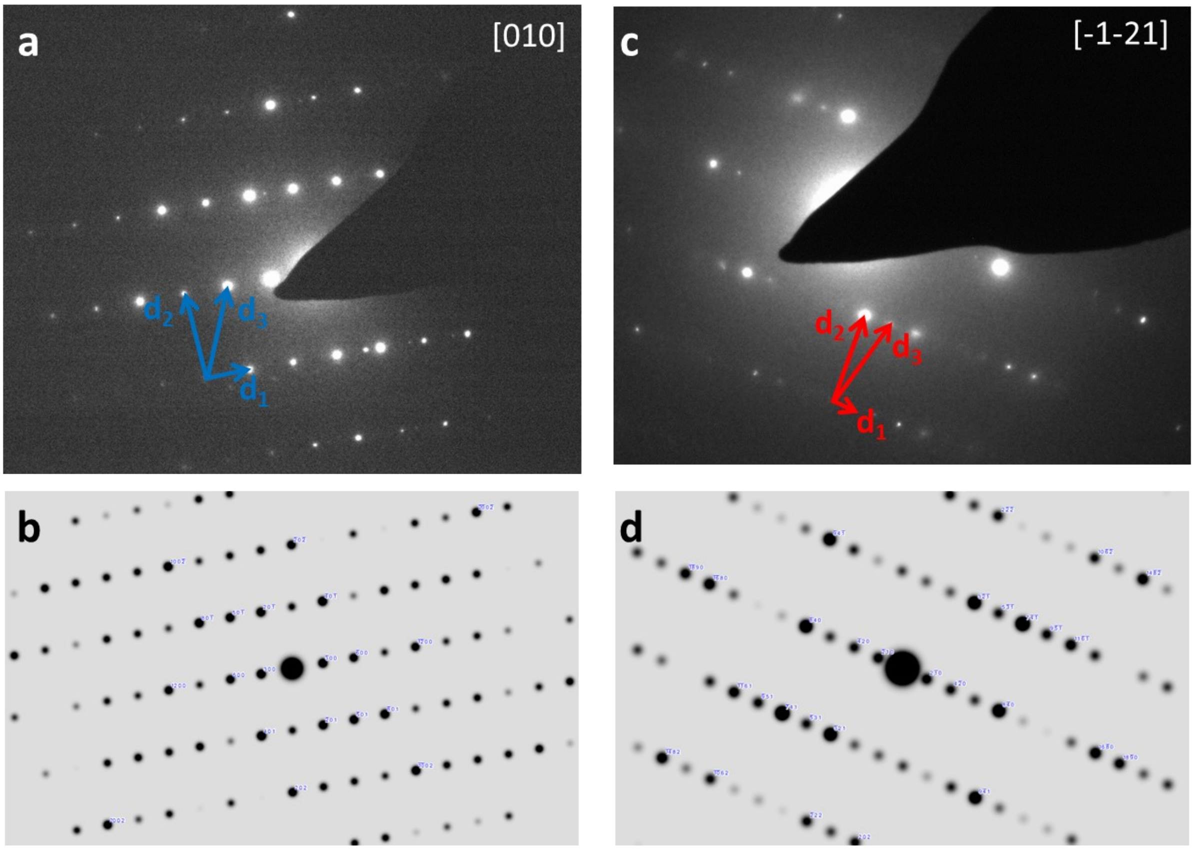

Figure S4 – Indexing of zone axis diffraction patterns of carbamazepine Form II (diffraction patterns obtained for samples of crystals of anhydrous CBZ Form II were of significantly better quality in comparison to data collected for CBZ·2H_2O under similar conditions). a) Experimental zone axis electron diffraction pattern of the [010] zone axis. b) Simulated electron diffraction pattern of the [010] zone axis from CSD structure CBMZPN03 (a = b = 35.454 Å, c = 5.253 Å, α = β = 90°, γ = 120°). c) Experimental zone axis electron diffraction pattern of the [-1-21] zone axis. d) Simulated electron diffraction pattern of the [-1-21] zone axis from CSD structure CBMZPN03.

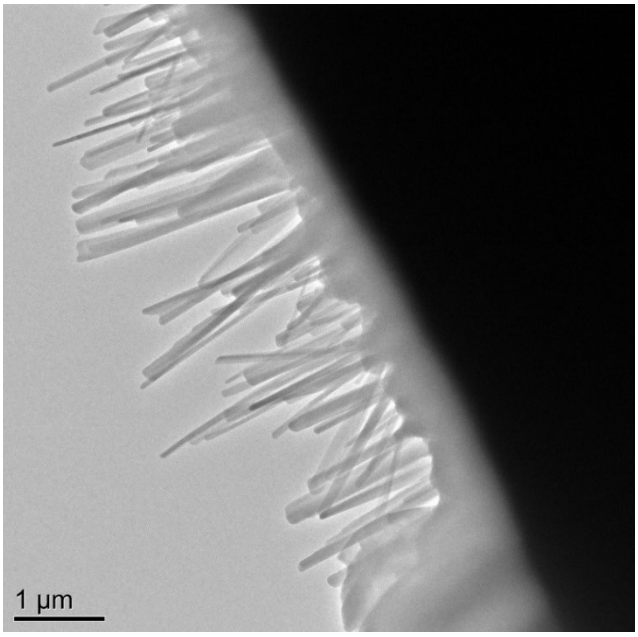

Figure S5 – TEM image of crystals of carbamazepine Form II. The bright field images confirm the absence of pores regardless of the time inside the vacuum chamber.

Please do not adjust margins

Please do not adjust margins

Sample identification – additional SEM images of CBZ·$2H_2O$

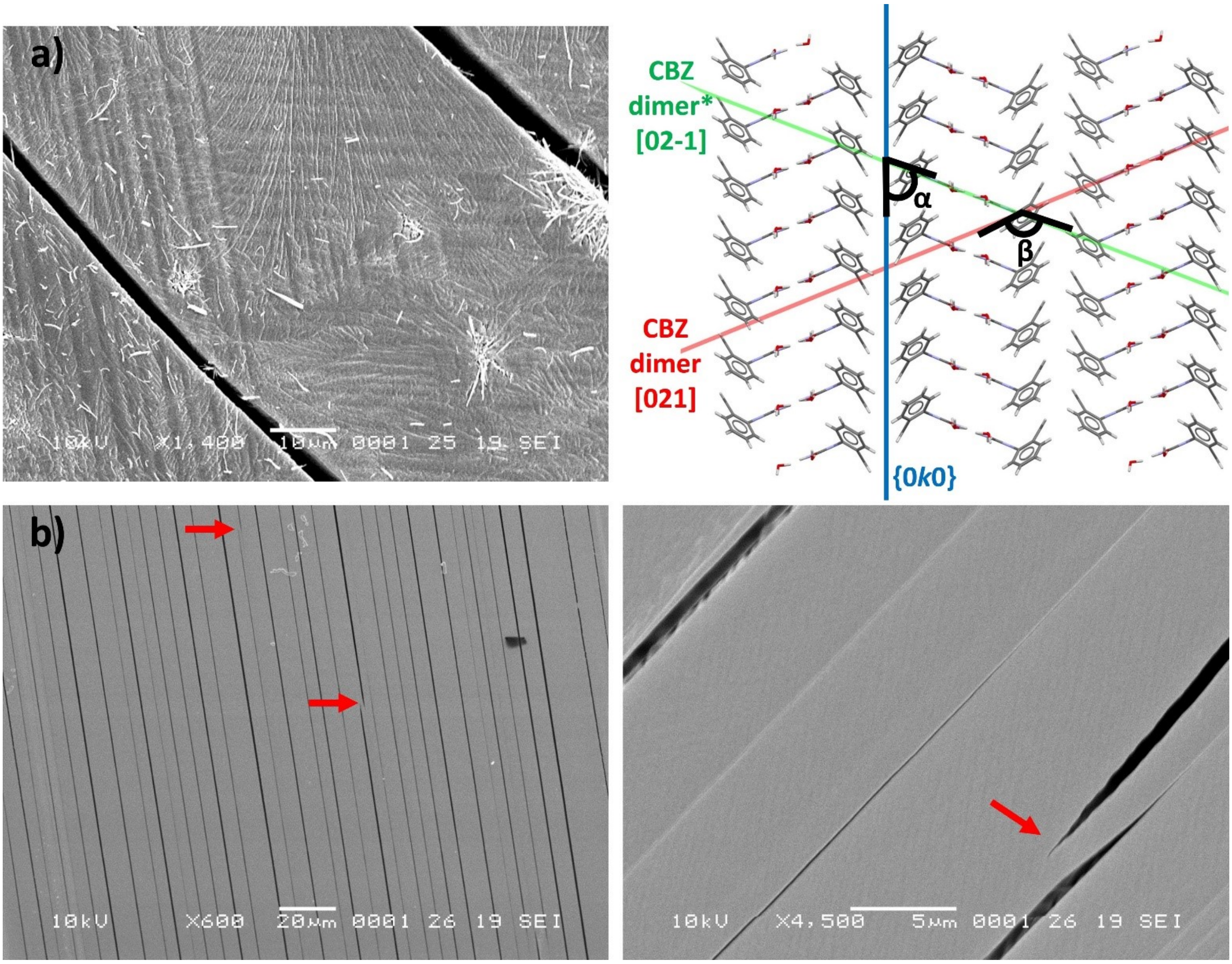

Figure S6 – Additional surface features on {*h*00} surfaces of CBZ·$2H_2O$. a) SEM micrograph of a dehydrated sample showing texture running across the cracks (left) and the molecular features associated to the textured domains (right). Angles between (020) planes and CBZ dimers and between pairs of CBZ dimers are represented by α and β, respectively. More texture images are available in **Figure S7**. b) incomplete crack propagation as highlighted by the arrows.

 This journal is © The Royal Society of Chemistry 20xx

Please do not adjust margins

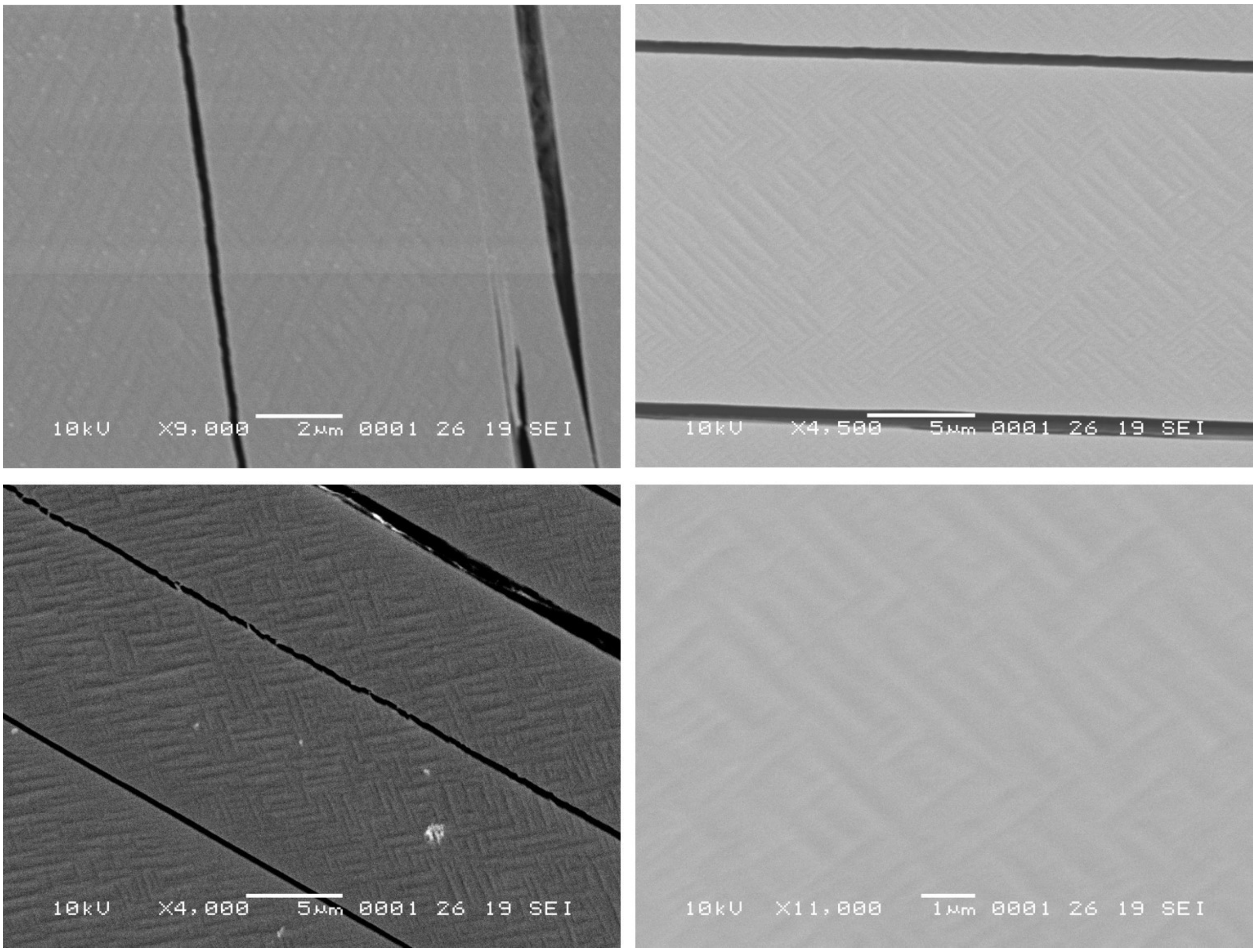

Figure S7 – SEM micrographs of the {h00} surfaces of CBZ·2H_2O fresh crystals showing its texture in detail.

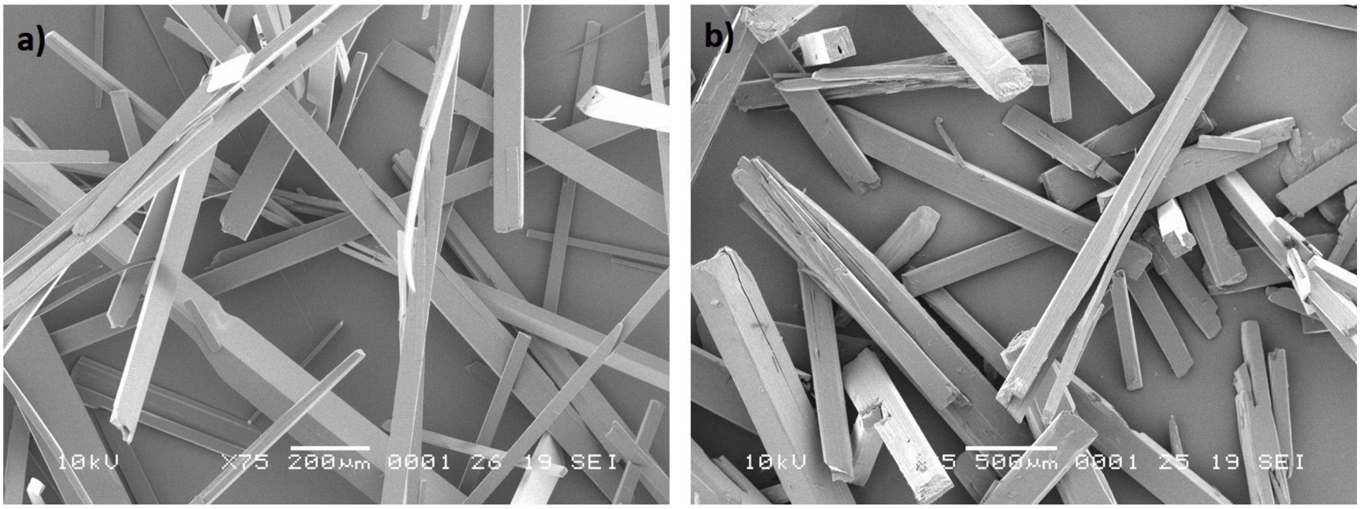

Figure S8 – SEM micrographs of CBZ·2H_2O prepared in laboratory scale and used in the general characterisation of crystal breakage as a consequence of vacuum and thermal treatment. The sample shown in **a)** presents {0*k*0} preferred surfaces, while the sample shown in **b)** presents {*h*00} dominant surfaces. See Experimental Section for details about the crystallisation conditions.

This journal is © The Royal Society of Chemistry 20xx

Please do not adjust margins

Please do not adjust margins

Transmission electron microscopy (TEM) of 2CBZ·BZQ

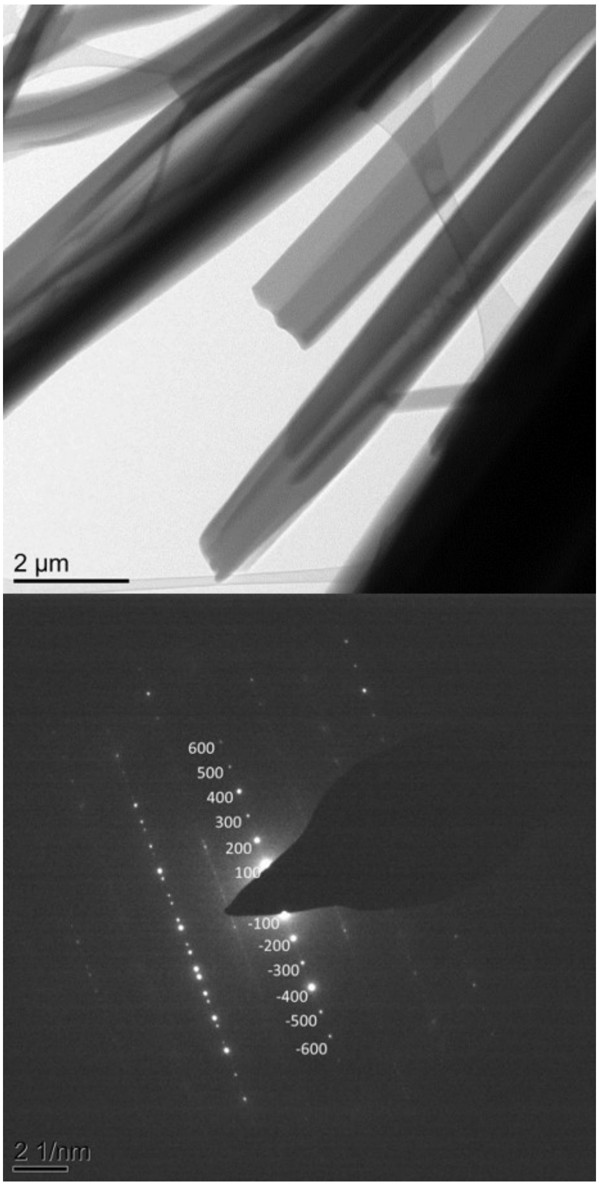

Figure S9 – TEM bright field image and diffraction pattern of 2CBZ·BZQ crystals (based on CSD structure UNEYOB, a = 10.334 Å, b = 27.612 Å, c = 4.996 Å, α = γ = 90°, β = 102.275°). The light/dark interfaces and the characteristics of the diffraction spots indicate twinning on (100).

This journal is © The Royal Society of Chemistry 20xx

Please do not adjust margins

Please do not adjust margins

Transmission electron microscopy (TEM) of CBZ·2H_2O

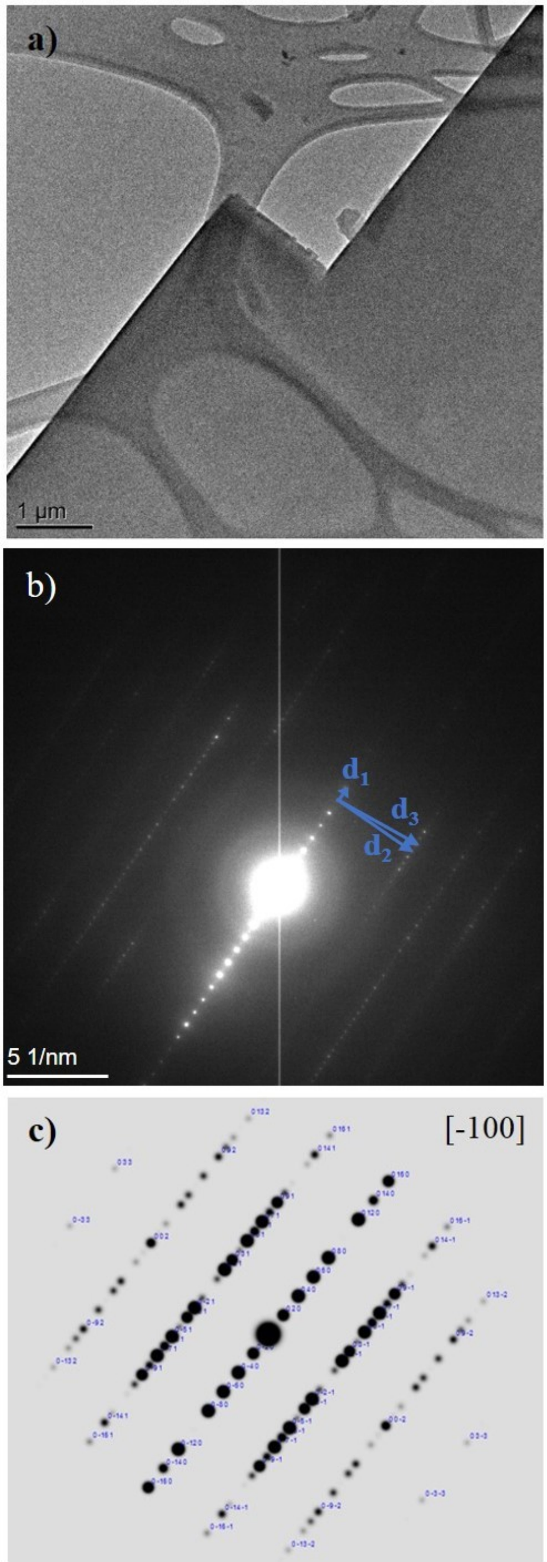

Figure S10 – TEM bright field image and diffraction pattern of CBZ·2H_2O obtained from cryo-TEM experiments. *a)* Bright field image. *b)* Respective experimental zone axis electron diffraction pattern. The [100] zone axis is shown in the simulated electron diffraction pattern (*c*) based on the CSD structure FEFNOT02 (a=10.066 Å, b=28.719 Å, c=4.831 Å, α=γ=90°, β=103.45°). The additional rows of spots seen in the experimental diffraction pattern in comparison to the simulated pattern suggest the presence of defects along the *b* direction.

Please do not adjust margins

Please do not adjust margins

Sample identification – PXRD

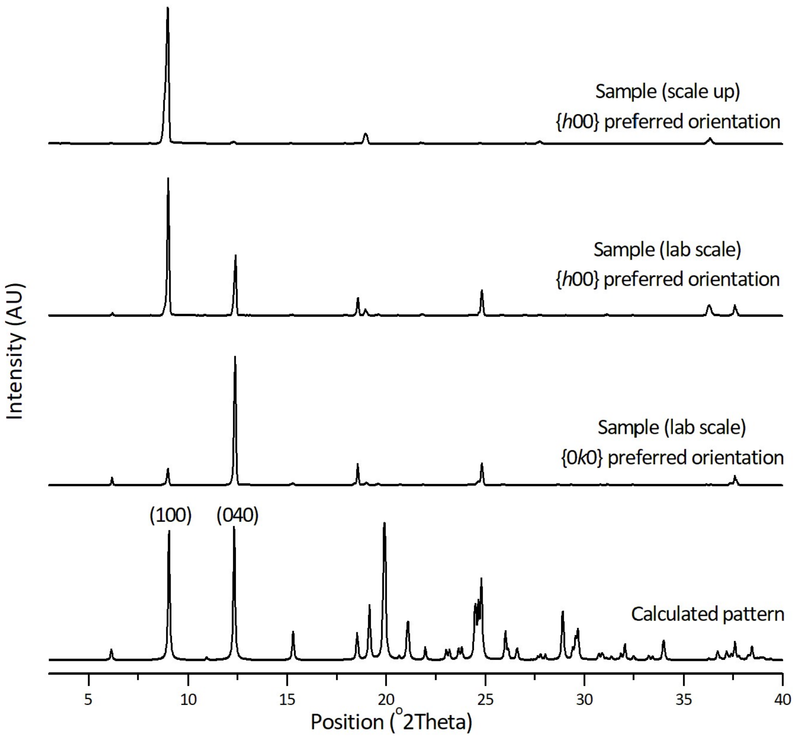

Figure S11 - PXRD patterns illustrating the preferred orientation of CBZ·2H_2O samples compared to the pattern calculated from FEFNOT02. While the samples prepared in laboratory scale were used in the general characterisation of crystal breakage because of vacuum and thermal treatment, the sample prepared in large scale was used in impact tests. See Experimental Section for details about the crystallisation conditions.

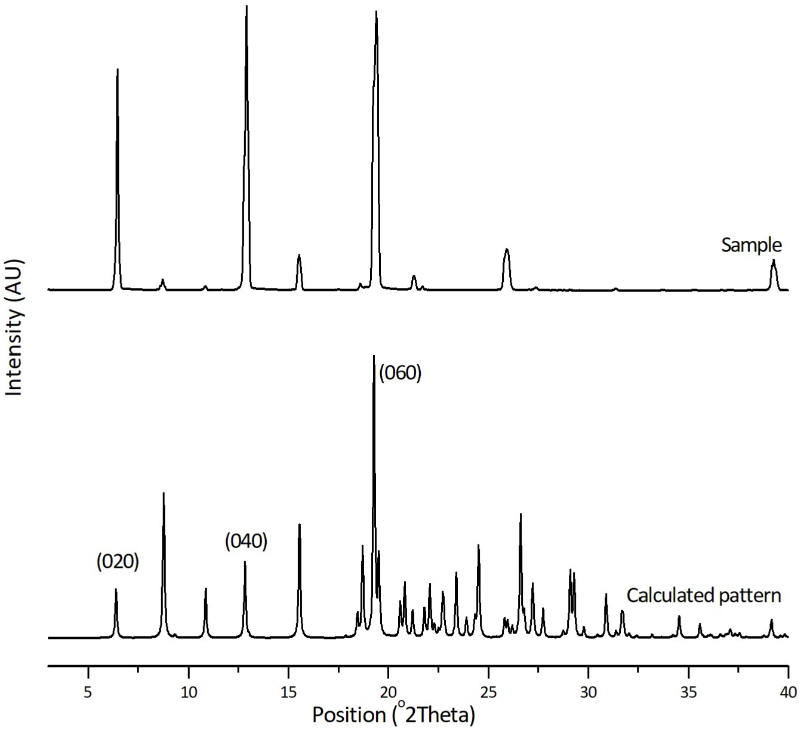

Figure S12 - PXRD patterns illustrating the preferred orientation of the 2CBZ·BZQ sample compared to the pattern calculated from UNEYOB. See Experimental Section for details about the crystallisation conditions.

 This journal is © The Royal Society of Chemistry 20xx

Please do not adjust margins

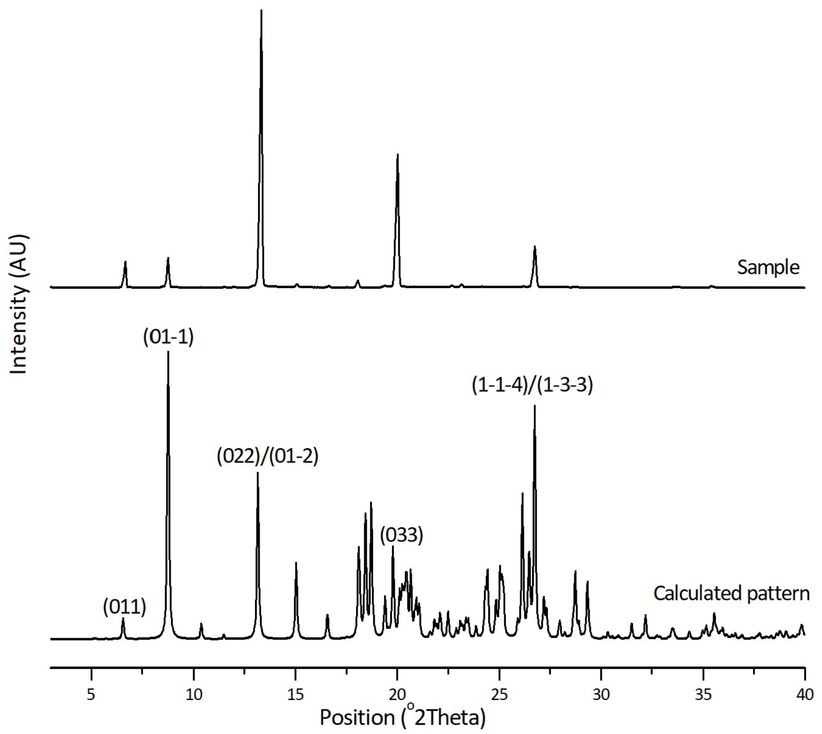

Figure S13 - PXRD patterns illustrating the preferred orientation of the CBZ·FORM sample compared to the pattern calculated from UNIBOI. See Experimental Section for details about the crystallisation conditions.

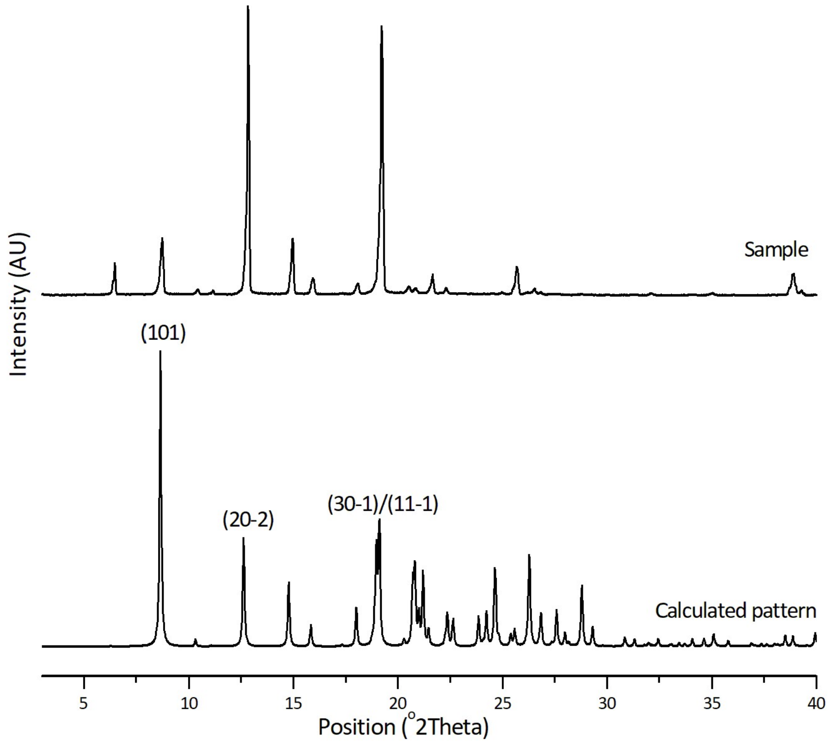

Figure S14 - PXRD patterns illustrating the preferred orientation of the 2CBZ·DIOX sample compared to the pattern calculated from QABHOU. See Experimental Section for details about the crystallisation conditions.

This journal is © The Royal Society of Chemistry 20xx

Please do not adjust margins

References

1 R. K. Harris, P. Y. Ghi, H. Puschmann, D. C. Apperley, U. J. Griesser, R. B. Hammond, C. Ma, K. J. Roberts, G. J. Pearce, J. R. Yates and C. J. Pickard, *Org. Process Res. Dev.*, 2005, **9**, 902–910.

2 S. G. Fleischman, S. S. Kuduva, J. a. McMahon, B. Moulton, R. D. Bailey Walsh, N. Rodríguez-Hornedo and M. J. Zaworotko, *Cryst. Growth Des.*, 2003, **3**, 909–919.

3 G. Schneider-Rauber, A. D. Bond, R. Ho, N. Nere, S. Bordawekar, A. Y. Sheikh and W. Jones, *Cryst. Growth Des.*, 2020, **21**, 52–64.

4 M. Ali, T. Bonakdar, M. Ghadiri and A. Tinke, *Powder Technol.*, 2015, **285**, 138–145.

5 T. Bonakdar, M. Ali, S. Dogbe, M. Ghadiri and A. Tinke, *Int. J. Pharm.*, 2016, **501**, 65–74.

6 S. Saifoori, W. P. Goh, M. Ali and M. Ghadiri, *Powder Technol.*, 2020, **361**, 651–662.

7 W. Pin Goh, M. Ali, K. Sinha, N. K. Nere, R. Ho, S. Bordawekar, A. Sheikh and M. Ghadiri, *Int. J. Pharm.*, 2019, **572**, 118780.

 This journal is © The Royal Society of Chemistry 20xx

Please do not adjust margins
